# Supplementary material for: Iterative improvement in the automatic modular design of robot swarms
Source: PeerJ Comput Sci. 2020 Dec 7;6:e322. doi: 10.7717/peerj-cs.322 (PMC7924708; doi:10.7717/peerj-cs.322)
Supplement: Supplemental Information 3 [file peerj-cs-06-322-s003.zip › argos3/doc/api/standalone/a00548_source.html]

ARGoS: plugins/robots/mini-quadrotor/control\_interface/ci\_miniquadrotor\_trajectory\_actuator.cpp Source File


- Main Page
- Related Pages
- Namespaces
- Classes
- Files

- File List
- File Members

# plugins/robots/mini-quadrotor/control\_interface/ci\_miniquadrotor\_trajectory\_actuator.cpp

Go to the documentation of this file.

```
00001 
00007 #include "ci_miniquadrotor_trajectory_actuator.h"
00008 
00009 namespace argos {
00010 
00011    /****************************************/
00012    /****************************************/
00013 
00014    CCI_MiniQuadrotorTrajectoryActuator::SWaypoint::SWaypoint() {}
00015 
00016    /****************************************/
00017    /****************************************/
00018 
00019    CCI_MiniQuadrotorTrajectoryActuator::SWaypoint::SWaypoint(const CVector3& c_position,
00020                                                              const CRadians& c_yaw) :
00021       Position(c_position),
00022       Yaw(c_yaw) {}
00023 
00024    /****************************************/
00025    /****************************************/
00026 
00027    void CCI_MiniQuadrotorTrajectoryActuator::CreateLuaState(lua_State* pt_lua_state) {
00028    }
00029 
00030    /****************************************/
00031    /****************************************/
00032 
00033 }
```

---

Generated on 10 Jul 2018 for ARGoS by 
 1.6.1
